# Management of a pregnant patient with chylomicronemia from a novel mutation in *GPIHBP1*: a case report

**DOI:** 10.1186/s12884-020-02965-1

**Published:** 2020-05-06

**Authors:** Min-Huan Lin, Xiao-Hui Tian, Xiu-Lan Hao, Hui Fei, Jian-Lan Yin, Dan-Dan Yan, Tian Li

**Affiliations:** Department of Obstetrics & Gynecology, the Seventh Affiliated Hospital of Sun Yat-Sen University, 628 Zhenyuan Road, Guangming District, Shenzhen, China

**Keywords:** GPIHBP1, Chylomicronemia, Hypertriglyceridemia, Pancreatitis, Pregnancy

## Abstract

**Background:**

Familial chylomicronemia syndrome (FCS) is a rare autosomal recessive lipid disorder often associated with recurrent episodes of pancreatitis. It is documented in most cases with FCS due to the mutations of key proteins in lipolysis, including LPL, APOC2, APOA5, LMF1 and GPIHBP1.

**Case presentation:**

We report the successful management of a 35-year-old pregnant woman carrying a novel homozygous frameshift mutation c.48_49insGCGG (p.P17A fs*22) in the *GPIHBP1* gene with previous severe episodes of acute pancreatitis triggered by pregnancy, resulting in adverse obstetrical outcomes. With careful monitoring, the patient underwent an uneventful pregnancy and delivered a baby with no anomalies.

**Conclusions:**

The case report contributes to the understanding of GPIHBP1-deficient familial chylomicronemia syndrome (FCS) and highlights gestational management of FCS patient.

## Background

Familial chylomicronemia syndrome (FCS), namely type 1 hyperlipoproteinemia according to Frederickson classification, is a rare disease with an incidence of about 1/1000,000 [1], yet is often complicated by recurrent and potentially fatal pancreatitis. The hallmark of FCS is abundant accumulations of chylomicrons in the circulation, lipemic appearance of patients’ serum, fasting triglyceride levels exceeding 1000 mg/dl (11.2 mmol/L) [2]. Typical manifestations of chylomicronemia include failure to thrive in infancy, recurrent pancreatitis in adolescence or adulthood, and physical examination and imaging may reveal hepatomegaly, splenomegaly, lipemia retinalis, cutaneous or visceral xanthomas, or coronary artery disease (CAD) [3].

So far as studies have revealed, FCS is often caused by homozygous mutations of some pivotal genes encoding key proteins in lipolysis, including *LPL* in the majority, as well as *APOC2*, *APOA5*, *LMF1* and *GPIHBP1* [4]. In contrast with FCS, acquired chylomicronemia is caused by LPL or GPIHBP1 autoantibodies [5]. GPIHBP1 was first identified in 2003 [6] and has been gradually recognized to play a critical role in lipolysis since 2007 [7]. There has been some published literature proving the lipolytic mechanism of GPIHBP1 and case reports of *GPIHBP1* mutations as well, but knowledge about GPIHBP1-deficient disease is still limited. We report a FCS patient, who had mild hypertriglyceridemia but recurrently exacerbated by pregnancy, causing severe pancreatitis that resulted in adverse obstetrical outcomes, one fetal demise and one child with cerebral palsy. Genetic sequencing reveals a homozygous frame-shift mutation of *GPIHBP1*, p.P17A fs*22. We also discussed the mutations in *GPIHBP1*, the influence of FCS towards pregnancy and the management in pregnant patient.

## Case presentation

A 35 year-old patient, gravida 4 para 1, was seen in our clinic at 9 weeks’ gestation age with a history of recurrent pancreatitis. Splenomegaly was found on routine physical examination when the patient was 23 years old. The initial attack of pancreatitis resulted in fetal demise at 22 weeks of her first pregnancy 7 years ago. The next attack occurred during her second pregnancy 4 years ago presented at 26 weeks’ gestation, after treatment it subsided gradually. The following third attack occurred at 35 weeks complicated by multiple organ failure, an emergency cesarean section was conducted and a female neonate delivered with severe asphyxia, later was diagnosed with cerebral palsy. The patient’s platelet count fell to 40 × 10^9^/L during the second pregnancy, with the level of 70–80 × 10^9^/L at postpartum. The fourth pancreatitis attack occurred 2 years ago was mild. The patient had no history of gallstones, alcohol use, diabetes mellitus or medication use. The parents and two siblings of the patient were all healthy, with no known consanguinity marriage in the family.

At her first visit to our clinic at 9 weeks’ gestation, the fact that all the episodes of pancreatitis were triggered by taking fatty food was noted; the suspicious hyperlipidemic pancreatitis was diagnosed although without the laboratory test. The patient’s weight was 47 kg, height was 160 cm, and body mass index (BMI) was 18.4 kg/m^2^ before pregnancy. No xanthomas or lipemia retinalis was found. Abdominal ultrasound revealed splenomegaly but no hepatomegaly. The plasma triglyceride level was 2.64 mmol/L, a high-density lipoprotein cholesterol level 0.70 mmol/L and a cholesterol level 2.58 mmol/L. She was recommended a low-fat diet. Plasma lipid levels were monitored monthly, later bimonthly, and showed a gradual increase of triglyceride and cholesterol levels. At 18 weeks’ gestation, when the plasma triglyceride level was 13.91 mmol/L and cholesterol 6.75 mmol/L, fenofibrate (200 mg once daily) was started to reduce the triglyceride level and decrease the risk of pancreatitis, with minimal effect.

**Fig. 1:**
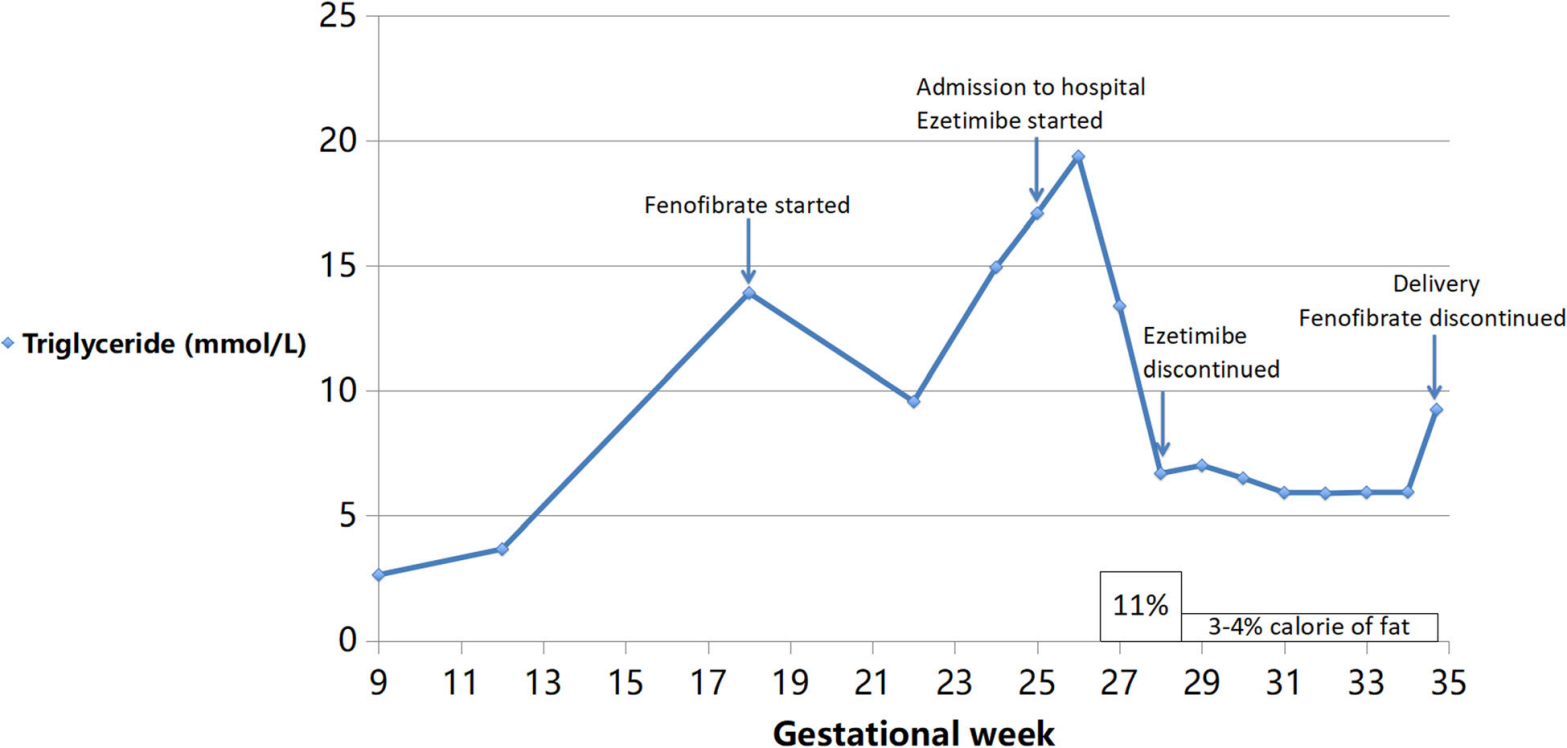
Triglyceride levels (shown as points connected by a line), percentage of dietary fat of total calories (shown as bars) and management of the patients at different gestational weeks

**Fig. 2:**
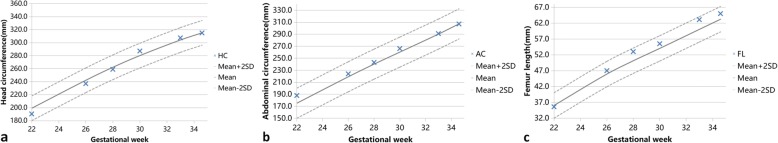
Fetal growth curve of the patient. With reference to fetal biometry in ethnic Chinese published by Leung TN et al. from Department of Obstetrics and Gynecology, Chinese University of Hong Kong

At 25 weeks, the patient was admitted to the obstetrical ward with a plasma triglyceride level of 17.09 mmol/L and a cholesterol level of 7.53 mmol/L (triglyceride levels throughout the pregnancy shown in Fig. 1). A multidisciplinary team consisting of obstetricians, dieticians, general surgeons and internal medicine specialists was involved. Combining the patient’s history of recurrent pancreatitis and blood test results, chylomicronemia was diagnosed. A low-fat diet was provided to the patient with 11% of total calories from fat per day since admission, dieticians formulated detailed diet plans including specific food of specific weight and the cooking method was water-boiling without cooking oil; besides fenofibrate 200 mg once daily, she was placed on ezetimibe 10 mg once daily and a pregnancy-supplement tablet once daily (containing 200 mg docosahexaenoic acid and 80 mg eicosapentaenoic acid). Although omega-3 fatty acids were recommended by the American Association of Clinical Endocrinologists (AACE) to lower triglyceride [8], it was not administered to our patient due to a lack of approval in China. Magnetic resonance of the pancreas showed no signs of post-pancreatitis complications. With dietary adjustment later on with only 4% of calories from fat per day, plasma triglyceride levels were slowly decreasing and ezetimibe was discontinued at 27^5/7^ weeks. Since 31 weeks’ gestation, the plasma triglyceride level was maintained between 5.64–5.98 mmol/L but could not be lowered any further. Monthly ultrasound showed normal growth of the fetus (fetal growth curve [9] shown in Fig. 2) and daily non-stress tests from 32 weeks did not yield abnormal results.

Taking into account the adverse pregnancy outcome of the patient and the knowledge of some acute pancreatitis occurrence with plasma triglyceride levels between 5.65 mmol/L to 11.3 mmol/L [10], it was recommended to perform cesarean delivery at 34^5/7^ weeks. A single course of corticosteroids was administered 48 h before the operation. On the surgery day, plasma triglyceride increased to 8.02 mmol/L, indicating the influence of dexamethasone toward triglyceride metabolism. A healthy male neonate weighing 2500 g was born, with Apgar scores of 9 and 9 at 1 and 5 min, respectively. Fenofibrate was discontinued post-partum, and she was discharged 8 days postpartum (plasma triglyceride level 6.06 mmol/L, body weight 58 kg). Keeping a low-fat diet with 6% calories from fat, until the latest follow-up (one hundred days postpartum), the patient’s plasma triglyceride at no time exceeded 10 mmol/L.

The neonate was admitted to Neonatal Intensive Care Unit (NICU) due to prematurity and was soon administered intravenously with lipid emulsion to correct the essential fatty acid deficiency. Although he suffered from recurrent apnea that required continuous positive airway pressure (CPAP) for 3 days, the neonate recovered well and was discharged on day 22. For the first one hundred days, the infant showed normal development physically.

Whole blood samples of the patient were sent to Beijing Genomics Institute-Shenzhen. The genomic DNA was extracted following standard protocols. The 3583 target genes for whole exome sequencing were based on Mendelian disease-causing variants from OMIM database (updated Jan 11th, 2016). Using the method of target capture and next-generation sequencing, the exons with intron-exon boundaries of target genes were sequenced in a BGISEQ-500 sequencer (MGI, Shenzhen). A total of 17,129.07 Mb of sequence data were produced with an average depth of coverage of 99.76 times and 95.89% of the total bases covered at 30 times. The result reveals a homozygous *GPIHBP1* frame-shift mutation c.48_49insGCGG (p.P17A fs*22), which was absent from the ExAC database. The insertion of 4 nucleotides at the codon 17 in the signal peptide domain of GPIHBP1 leads to the introduction of the stop codon at the position 22, probably producing a truncated and nonfunctional protein. Combining results from the prediction tools Condel [11], PolyPhen2 and SIFT, this mutation is considered likely pathogenic according to the guidelines of the American College of Medical Genetics and Genomics.

## Discussion and conclusions

We describe a patient with GPIHBP1-deficient FCS who had had adverse pregnancy outcomes due to severe pancreatitis. A stringent low-fat diet and oral fenofibrate were able to decrease triglyceride at a relatively safe level. In addition, we closely monitor the patient’s liver enzyme, creatine kinase, blood glucose and fetus NST and growth. Although in our case ezetimibe was advised by the internal medicine specialist to lower cholesterol, it wasn’t recommended in FCS cases in literature. Given the adverse pregnancy outcome of the patient and the risk of pancreatitis, we delivered the baby at 34^5/7^ weeks through cesarean section. Though corticosteroids was administered for fetal maturation, it did trigger synthesis of triglyceride and might have the risk of triggering pancreatitis, reminding us to cautiously use corticosteroids on FCS patients. In all, the pregnancy outcome is favorable.

Of note, FCS is characterized by accumulations of chylomicrons in the circulation. The synthesis of chylomicrons is the first step of the exogenous triglyceride lipolytic process [12]. Triglyceride-rich lipoproteins (TRLs), constituted by chylomicrons and very low-density lipoproteins (VLDLs), are processed by lipoprotein lipase (LPL) with other cofactors including glycosylphosphatidylinositol-anchored high density lipoprotein-binding protein 1 (GPIHBP1) [13]. GPIHBP1 is a glycosylphosphatidylinositol-anchored glycoprotein of the Lymphocyte Antigen 6 (Ly6) family specifically generated in capillary endothelial cell [13].There are four exons of the GPIHBP1 encoding four key features, including an amino-terminal signal peptide, an acidic domain, a three-fingered LU (Ly6-uPAR/lymphocyte antigen 6-urokinase-type plasminogen activator receptor) domain and a signal sequence [14]. After modification of GPIHBP1, it is trafficked to both the basolateral and apical surface of endothelial cells [12], mainly in skeletal muscle, heart and adipose tissue, being virtually absent in the brain [7]. GPIHBP1 actively transports LPL from interstitial space to capillary lumen and the GPIHBP1-LPL binding “attenuates the unfolding of LPL’s catalytic domain” [14], thus prompting LPL to process TRLs in the capillary. With deficient *GPIHBP1* gene, including homozygous or compound heterozygous missense mutations, GPIHBP1 is polymerized and chylomicrons accumulate in the circulation, causing FCS [15]. Plus, mutations in *LPL*, *APOC2*, *APOA5* and *LMF1* genes cause FCS as well, while LPL or GPIHBP1 autoantibodies lead to acquired chylomicronemia [5]. Strangely enough, *Lpl*-kicked out mice died shortly after birth with plasma triglyceride levels as high as 20,000 mg/dl [16], whereas *Gpihbp1*-kicked out mice survived and had far lower triglyceride levels (plasma triglyceride levels of adult mice 1000–5000 mg/dl, pup mice 120 ± 12 mg/dl) [7]. Such phenotypic differences from LPL-deficient cases were allegedly attributed to “the fenestrated capillaries of the liver which allow access of TRLs to LPL” [14] even without properly functioning GPIHBP1.

FCS has come to be recognized as one of the leading factors of acute pancreatitis in pregnancy. There is physiologic hypertriglyceridemia occurring during the last two-thirds of gestation due to the progressive climb of plasma estrogen levels and the insulin-resistant condition, both enhancing VLDL production and decreasing LPL expressions and activities in the liver [17]. In uncomplicated pregnancies, by the third trimester, plasma triglyceride levels rise between 2- and 4-fold, with a mean of 79 mg/dL, 151 mg/dL and 245 mg/dL in the first, second and third trimester respectively [18]. In severe hypertriglyceridemia especially in FCS cases, large amounts of triglycerides in the pancreas are hydrolyzed by pancreatic lipase, producing a vast number of free fatty acids (FFA), which causes injury to the pancreatic acinar cells and capillaries. In such an acid environment, pancreatic local ischemia and activation of trypsinogen by FFA trigger acute pancreatitis (Havel et al., 1969) [19].

Well-recognized guidelines of FCS in the non-pregnant state have been published [2, 8, 20], but few for the pregnant population [21]. Based on our experience with this patient and literature studies, for pregnant women with or without FCS, baseline lipid profiles should be evaluated at early pregnancy for better supervision during pregnancy. For patients with chylomicronemia, a nuanced approach should be developed to balance between maternal triglyceride control and fetal needs. A strict low-fat diet formulated by a dietician is the mainstay therapy of FCS. Theoretically, seeing that chylomicrons are produced exogenously [12], a strictly formulated low-fat diet (less than 20% of total calories from fat per day, or furthermore, less than 10%) should be effective in reducing triglyceride levels [21]. Intriguingly, the risk of macrosomia and large for gestational age (LGA) is positively correlated with maternal serum triglyceride levels [22]. In our case, even with low-fat diet, fetal growth was appropriate for the gestational age. To avoid essential fatty acid deficiency, omega-3-acid ethyl esters and medium-chain triglycerides (MCT) are also recommended as additional nutritional support [8, 23]. If a patient’s not compliant to the diet or triglyceride levels are refractory to control, hospitalization should be considered for better diet supervision [24]. Fibrates have been reported in quite some studies to effectively control triglyceride levels without fetal malformation or infant development [24, 25]. Fasting, parenteral nutritional support, insulin infusion are routinely needed with pancreatitis, or in some cases without pancreatitis [26]. Plasmapheresis is proven effective in refractory cases [27, 28]. If early induction of labor or preterm C-section is required in intractable situations, obstetricians should be prudent to prescribe dexamethasone for use of fetal maturation, since dexamethasone might trigger pronounced triglyceride increase or pancreatitis.

In conclusion, this case report contributes to the understanding of GPIHBP1-deficient familial chylomicronemia syndrome (FCS). We should be aware of potential dyslipidemia in pregnant women even without a family history. We also highlight lipid evaluation at early pregnancy, diet formula and gestational management for patients with FCS.

## Data Availability

The datasets analyzed during the current study are not publicly available due to protection of the patient’s privacy but are available from the corresponding author on reasonable request.

## Abbreviations

FCS: Familial chylomicronemia syndrome
CAD: Coronary artery disease
BMI: Body mass index
AACE: American Association of Clinical Endocrinologists
NICU: Neonatal Intensive Care Unit
CPAP: Continuous positive airway pressure
TRLs: Triglyceride-rich lipoproteins
VLDLs: Very low-density lipoproteins
LPL: Lipoprotein lipase
GPIHBP1: Glycosylphosphatidylinositol-anchored high density lipoprotein- binding protein 1
Ly6: Lymphocyte Antigen 6
LU: Ly6-uPAR/lymphocyte antigen 6-urokinase-type plasminogen activator receptor
FFA: Free fatty acids
LGA: Large for gestational age
MCT: Medium-chain triglycerides
